# The Role of Innate Lymphoid Cells in Cancer Development and Immunotherapy

**DOI:** 10.3389/fcell.2022.803563

**Published:** 2022-04-26

**Authors:** Rio Sugimura, Clement Yisai Wang

**Affiliations:** School of Biomedical Sciences, The University of Hong Kong, Pokfulam, Hong Kong SAR, China

**Keywords:** innate lymphoid cell, cancer immune cell therapy, tumor microenvironment, immune checkpoint, cytokines

## Abstract

Innate Lymphoid Cells (ILCs) are an elusive type of innate immune cell that was only discovered recently. Their tissue residency and dependency makes them a niche group of cells that bridge the adaptive and innate immune system. The nomenclature and classification of ILCs have been challenging due to their heterogeneity. The currently agreed ILC classification splits the cells into two categories including cytotoxic and helper ILCs. The tumour microenvironment is often hostile for immune cells. Remodeling the microenvironment and regulating other immune cells—achieved by ILCs-can enhance anti-tumor effects. How ILCs regulate other immune cells in the tumor microenvironment remains to be understood. Here we review current understanding of the role of ILCs in the tumor microenvironment. ILCs recruit CD8 positive T and memory T cells in PDAC, ILCs are also able to help CD108 positive B cells migrate toward tumour locations. In NSCLC, ILC3s are seen helping resident macrophages enhancing the mucus immunity to cancer cells. We then highlight the roles of cytokines and immune checkpoint pathways in ILCs and its implication in immunotherapy.

## 1 Introduction to Innate Lymphoid Cells

The human body’s innate and adaptive immune system has evolved to achieve a peaceful cohabitation with various microorganisms while protecting the body from foreign pathogens. This closely-knit immune “community” also contributes to homeostasis, tissue repairs, and a ray of other essential functions. Recently, with the revolutionary discovery of innate lymphoid cells (ILCs)—a new group of innate immune cells—we are seeing a brand-new collection of cells that communicate with both the innate and adaptive immune system, bridging the gaps between other immune cell functions, and orchestrate inflammatory responses throughout the body.

ILCs are a niche branch of the innate immune system sensitive to multiple cell-derived signals such as hormones, cytokines, and peptides (Artis and Spits, 2015). Innate lymphoid cells also depend on toll-like receptor (TLR) activation, responding to TLR ligands in autoimmune diseases such as asthma (Crellin et al., 2010). With the growing understanding of the heterogeneity of functions the different ILC subtypes perform, we realize the immense potential of ILCs in clinical environments. In this section, we will cover the classification, origin, and functions of ILCs to build up the distinction regarding the roles of ILCs in cancer development and immunotherapy.

### 1.2 Classification of ILC Subsets

**FIGURE 1:**
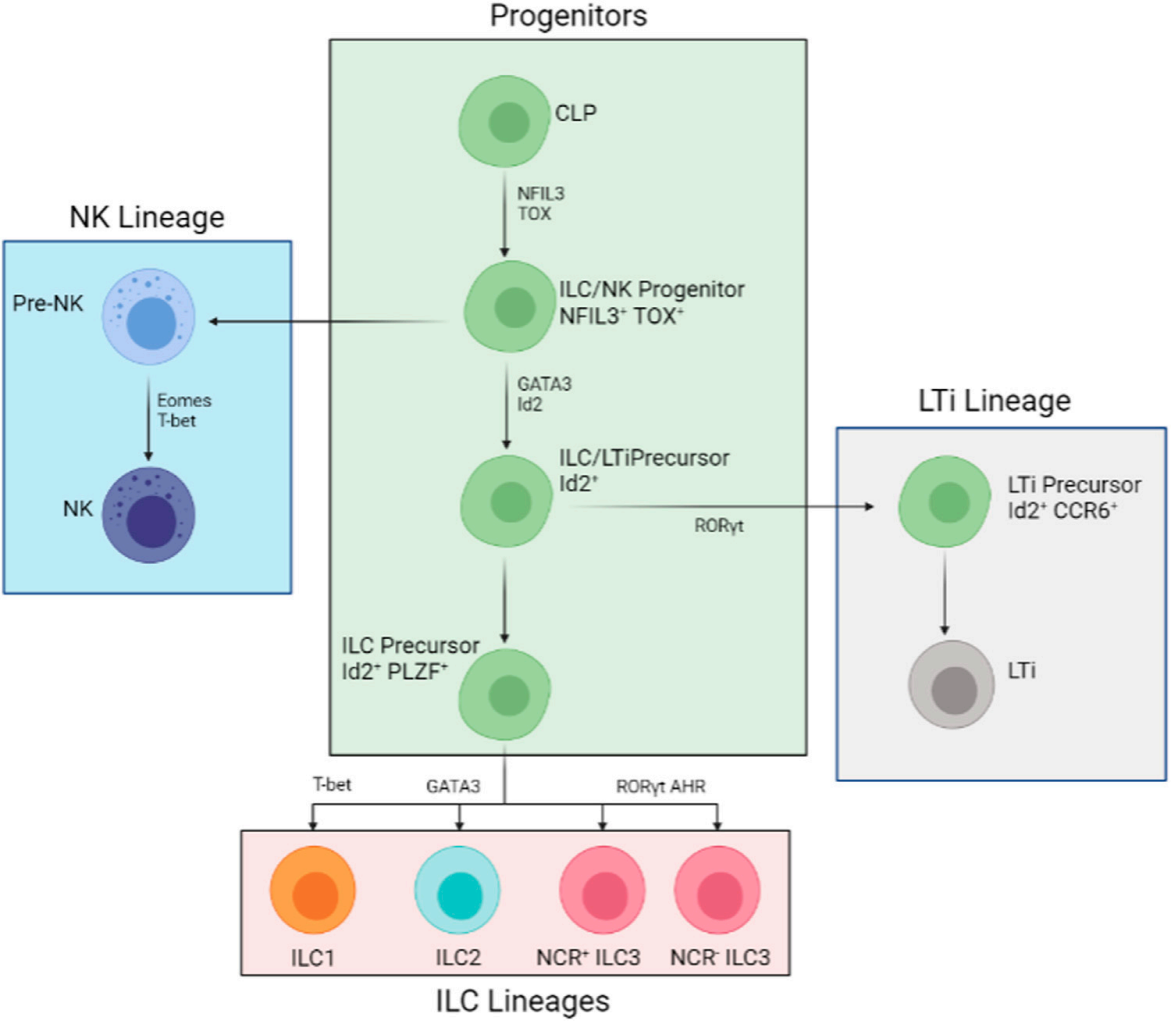
Developmental Process of the ILC Family. All members of the ILC family derive from the common lymphoid progenitor (CLP). The developmental pathways are mostly split into three lineages: NK lineage, ILC lineage, and LTi lineage. The NK pathway (in blue) is the first to derail from the main pathways (in green). The downstream progenitor is an Id2+ NFIL3+ TOX + LTi and ILC precursor and Id2+ NFIL3+ TOX + PLZF + precursor restricted to ILCs (in pink). The LTi restricted precursor (in grey) is an intermediate cell stage that is, Id2+ RORγt + CCR6+. Further down from the restricted ILC-only precursor, T-bet, GATA3, and RORγt AHR are needed for ILC1, ILC2, and all types of ILC3 development respectively.

ILCs categorizes into three subsets: NK/ILC1s, ILC2s, and ILC3/LTi (Lymphoid tissue inducers). ILC1s and NKs are also referred to as cytotoxic ILCs, while ILC2, ILC3, and LTi are known as helper ILCs, though some ILC3 cells are also capable of producing cytotoxins (Artis and Spits, 2015). All members of the ILC family follow similar lymphoid cell morphology, lacking cell-surface immune identification molecules and cell lineage markers (Lin-) (Spits and Di Santo, 2010). Alongside secretion (including IL-22 and IL-33) and reception of many cytokines (including IL-12R and IL-17R), members of the ILC family all share similar key gene expressions and transcription factors enhancements, examples of which include T-bet (T-cell associated TF), EOMES (Eomesodermin), and RORs (RAR-related orphan receptors) (Artis and Spits, 2015). Recent genomic analyses have shown that cytotoxic and helper ILCs are different cell lineages (Das et al., 2018; Mazzurana et al., 2021). These analyses revealed key transcriptome and surface marker differences between cytotoxic and helper. The following paragraph will discuss the differences of different ILC subtypes more in-depth (Figure 1).

**FIGURE 2:**
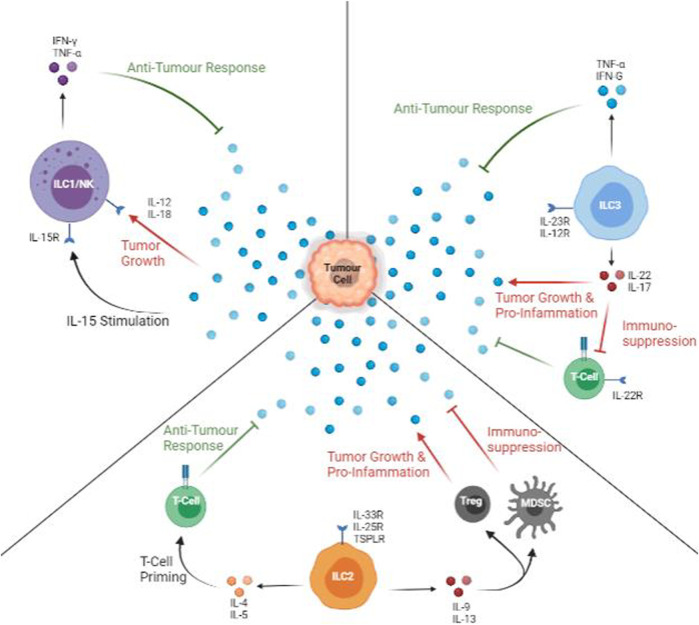
Anti-tumour and Pro-tumour Roles of ILCs. All types of ILCs can protect against (green arrows) and promote (red arrows) tumour growth. ILC1s are active via IL-15, and IFN-γ and TNF-α are released. In the presence of IL-12 or IL-18, ILC1 cytokine profile is decreased, facilitating tumour growth. ILC2s are stimulated mainly by IL-33, but it produces a mix of pro- and anti-tumour cytokines. IL-5 can aid in T-cell priming with tumour specific markers while IL-9 or IL-13 promote Treg and myeloid-derived suppressor cells, promoting inflammation and suppressing T-cells. The presence of IL-12 promotes anti-tumour cytokines. The presence of IL-23 promotes productions pro-tumour and suppressive cytokines.

Each subtype of ILC has its distinctive phenotypical features and cytokine profiles. ILC1s, similar to natural killer cells inside the human body, are Lin-, IFN-γ producing cells (Fuchs et al., 2013). Other secretions from ILC1s include TNF-α (tumor necrosis factor) and inflammatory factors that respond to intracellular bacteria and parasites such as perforin. ILC2, by contrast, produces mostly T-helper-cell-associated cytokines: IL-4, IL-5, and IL-13. Thus, rather than fighting off pathogens, ILC2s’ primary role is to recruit immune cells and bridge the gap between the innate and adaptive immune systems (Starkey et al., 2019). Finally, stimulus-dependent CCR6- ILC3s: splitting into natural cytotoxicity receptor (NCR or NKp46) positive and negative groups. The term NCR is used to distinguish NKp46 expression on NK cells and expressions on ILC cells. NCR + ILC3s express cytotoxic molecules that aid the adaptive immune system while NCR- ILC3s are more responsible for the homeostasis of microbes inside the human intestines and tissue repair (Figure 2) (Melo-Gonzalez and Hepworth, 2017). On the other hand, CCR6+ ILC3 are usually CCR6+ CD4^+^ LTi cells. LTi cells form and maintain lymph node structures (Withers, 2016). The previously mentioned classifications of innate lymphoid cells are based on function rather than their phenotype. Phenotypical classifications will only convolute the discussion of ILCs and their roles in cancer development (Spits et al., 2013).

### 1.3 Origin of Human ILCs

All members of the ILC family derive from the same type of progenitor, the common lymphoid progenitor (CLP). Phenotypically, these progenitors are classified as Lin- Id2+ IL-7R + CD25- α4β7+ cells, whilst are RORγt- T-bet- Eomes- (Artis and Spits, 2015) (Figure 1). Unlike progenitors committed to either T-cell or B-cell lineages, CLPs’ surface-bound markers suggest that they are less committed to one specific cell type. Thus, theoretically, CLPs should give rise to all members of the ILC family equally. There is evidence of hematopoietic precursors capable of generating all types of ILC (Constantinides et al., 2014; Harly et al., 2017). However, reports suggest the existence of ILC3 lineage committing CLPs inside the mouse bone marrow. These committed progenitors-derived ILC-like cells show potential transdifferentiation to migrate to peripheral tissues and peripheral blood streams. Although these committed progenitors are yet to be observed in humans, there is a great possibility that lineage committed progenitors do exist in main ILC generating locations. More insight is needed to determine the pathways and mechanisms of cell lineage committing CLPs.

Throughout the developmental process from CLPs to mature ILCs, several transcription factors are needed at different stages. During early differentiation, TOX and NFIL3 are crucial for all helper and cytotoxic ILC development (Aliahmad et al., 2010; Klose et al., 2014). Alongside Id2, which somewhat restricts cytotoxic ILC development *in vivo* conditions, TOX and NFIL3 promote the development of CLP into ILC2s and ILC3s (Harly et al., 2017). Further down the line, GATA-3 and TCF-1 are important for all helper ILCs (Withers, 2011; Diefenbach et al., 2014). Studies involving genetically removing GATA-3 from hematopoietic stem cells inhibited all ILCs and T cells except of B and NK cells (Klose et al., 2014). Furthermore, GFI1 is found to act upon Gata3 gene expression (Shao et al., 2021); deficiency in GFI1 gives rise to GATA3low ILC2 and ILC3 populations (Withers, 2011; Diefenbach et al., 2014). Later survival and functionality tests also revealed shorter survival and reduced cytokine production than GATA3high and normal ILC2 and ILC3 (Hoyler et al., 2012). On top of that, PLZF was found crucial for all helper cell maturation. ILC precursors that lack zinc finger proteins (from genetic knockouts) are unable to produce ILCs with mature surface markers and cytokine profiles (Artis and Spits, 2015). Thus, GATA3 and the two zinc finger proteins (GFI1 and PLZF) assist in the maturation in all helper ILC cell types. Specific genes are highlighted in different ILC types. For ILC1s, is T-bet; GATA3 is crucial for ILC2s; RORγt is important for both ILC3 and LTi differentiation (Diefenbach et al., 2014) (Figure 1 and Table 1).

**TABLE 1:** Cell surface molecules and transcription factors present on each type of ILCs.

Cell type		NK	ILC1	ILC2	NCR + ILC3	NCR- ILC3	LTi
Cell surface molecules	CD45	+	+	+	+	+	Weak
	CD127 (IL-7Ra)	heterogenous	+	+	+	+	+
	CD161 (-K1.1)	heterogenous	+	+	+	+	heterogenous
	ST2	heterogenous	-	heterogenous	-	-	-
	CD278 (ICOS)	-	-	+	+	-	heterogenous
	KLRG1	+	-	+	-	-	-
	CD117	heterogenous	-	heterogenous	+	+	+
	CD69	weak	heterogenous	N/A	N/A	N/A	N/A
	CD254 (RA-KL)	-	-	heterogenous	+	+	+
	CD196 (CCR6)	-	heterogenous	heterogenous	heterogenous	heterogenous	+
	CD335 (NK+46)	+	-	-	weak	weak	-
	CD25 (IL-2Ra)	+	weak	+	weak	weak	weak
	CD314 (NKG2D)	+	-	-	weak	weak	-
	CD194 (CCR4)	N/A	weak	+	N/A	N/A	N/A
	CD183 (CXCR3)	N/A	+	N/A	N/A	N/A	N/A
	CD56	+	-	-	weak	weak	-
Transcription Factors	Tbet	+	+	-	-	-	-
	Eomos	+	+	-	-	-	-
	ROR	-	weak	weak	+	+	+
	GATA3	weak	weak	+	+	weak	weak
	AhR	-	-	+	+	+	+
	ZBTB16	weak	weak	+	weak	weak	-
	NFIL3	-	weak	weak	weak	weak	weak
	TCR7	weak	weak	-	weak	-	-
	KIT	-	weak	weak	weak	weak	-
	PTGDR2	-	weak	weak	weak	-	-

Cell surface molecules (CSM) and transcription factors (TF) are listed against each type of ILCs. “+” symbol represents presence of CSM or TF, “-” symbol represents absence of CSM or TF, “heterogenous” markings represents variating levels of CSM and TF, “weak” markings represent positive but relatively weaker presentation of CSM or TF on ILCs.

### 1.4 Heterogeneity of ILC Functions

Tissue-resident innate lymphoid cells congregate in secondary lymphoid structures, mucosal barriers, and other barrier surfaces. Having a great chance of encountering pathogens, ILCs are programmed to respond to inflammatory signals, alarmins, and other cytokines, providing an essential function in the innate immune system (Artis and Spits, 2015). For example, IL-12, IL-15, and IL-18 stimulate NK cells and ILC1 class cells; IL-2, IL-4, IL-25, IL-33, and IL-9 induce ILC2 recruitment; IL-1β and IL-23 stimulate ILC3s (Spits and Di Santo, 2010; Artis and Spits, 2015; de Weerdt et al., 2016). In the context of immunity toward bacteria and parasites inside the human body, ILC1s and ILC3s are extremely important, especially in newborns where their adaptive immunity is still being built. For example, infections caused by *Salmonella enterica* and Cryptosporidium are treated by IFN-γ producing ILC1s (Kumar, 2014; Gullicksrud et al., 2021). Cytotoxin-producing ILC3s also help ward off gram-negative bacteria infection caused by *Citrobacter* rodentium (Gerbe et al., 2016). Furthermore, IL-22 produced by all classes of ILC3s is one of the critical cytokines (alongside lymphotoxins) that helps with intestine microbes homeostasis (Geiger et al., 2014; Goto et al., 2014).

Much current research on ILCs is conducted using non-human-derived cells. Thus, it is important to understand that non-human ILCs may be significantly different from human ILCs due to cytokine differences between species. In humans, NK lineage is more incorporated into the ILC progenitor whereas mouse progenitors are less capable of NK production (Scoville et al., 2019). ILC3s present the most heterogeneity between humans and mice. The difference is likely because ILC3s rely more on myeloid and epithelium cells to relay stimuli (Hoorweg et al., 2012).

## 2 Innate Lymphoid Cells During Cancer Development

Inflammation is a critical component of cancer as it plays a significant role in tumorigenesis. In addition, chronic inflammation promotes instability and angiogenesis in a local area, promoting tumour growth (Kashii et al., 1999). These inflammation conditions are caused by several reasons, such as smoking, obesity, and bacterial infections (de Martel and Franceschi, 2009). Innate lymphoid cells are present in most of these inflammatory cases. Innate lymphoid cells are usually present under normal and inflammatory conditions. Unlike NK or T cells, which are attracted into the inflamed regions, ILCs are activated locally and contribute to around 1–5 percent of all immune cells present, with a large portion of helper ILC cells (Hazenberg and Spits., 2014). In many cancers, such as in non-small-cell lung carcinoma (NSCLC), chronic lymphocytic leukaemia (CLL), and hepatocellular carcinoma (HCC), each type of innate lymphoid cells devote to a distinctive component of cancer immunity. In this section, we will investigate pro- and anti-tumorigenic roles of innate lymphoid cells in several cancers.

### 2.1 Anti-Tumour Role of ILC During Cancer Development

#### 2.1.1 In Lung Cancers

Lung resident ILC2s are functionally dependent on IL-33 levels in the local microenvironment. In immuno-challenged mice models with established orthotopic lung tumours (TC1), metastatic lung tumours (A9) arose soon after TC1 implantation (Saranchova et al., 2018). A9 appearance greatly reduces IL-33 prevalence and increases circulating tumour cells (CTCs). Unsurprisingly, ILC2 function and cytokine profile is greatly diminished. Later, when TC1 and A9 cell lines were implanted with ILC2 deficient mice, tumour growth increased significantly with more metastatic tumour particles to distal organs (Saranchova et al., 2018). Research by Carrega’s team also suggests NCR + lung resident ILC3 cells are able to interact with both lung tumour cells and tumour-associated fibroblasts (Carrega et al., 2015). Producing key anti-tumour and immune activation cytokines such as TNF-α, IL-8, IL-2, and IL-22, NKp46 activated ILC3s accumulate significantly in stage I/II in NSCLC patients. NCR + ILC3 accumulation in early stage lung cancer also correlates with higher density of intratumoral tertiary lymphoid structures (TLS), suggesting ILC3s crucial role on early cancer protections in the lungs (Carrega et al., 2015).

#### 2.1.2 In Melanoma and Breast Cancers

TCR−NK1.1 + CD49ahi cells type 1-like ILCs were discovered in murine mammary tumour bearing PyMT models (Ikutani et al., 2011). These novel ILC1-like cells differ from conventional ILC1 and NKs because they express both ILC1 phenotypes and granzyme B at the same time. In further experiments, these novel ILC1-like cells produced toxins within the tumour that inhibits tumour growth. IL-15 transgenic overexpression in the same PyMT models also further expanded TCR−NK1.1 + CD49ahi ILC1-like cells around tumour areas. Mice with IL-15 overexpression also display higher granzyme and anti-tumoural cytokine production, suggesting IL-15/ILC1-like cell antitumor axis in breast cancers (Ikutani et al., 2011). Furthermore, in murine lung metastatic melanoma cancer B16F10 model, IL-33 and IL-25 stimulated ILC2 help recruit eosinophil and maintain immune cell balance in the lung to prevent or minimise metastatic effect (Eisenring et al., 2010). Similarly, exogenous IL-5 treatment greatly suppressed tumor metastasis and induced eosinophil infiltration (Ikutani et al., 2011). When subcutaneous murine B16 melanoma is injected with IL-12, NCR + ILC3 cells exhibit tumor-suppressive function (similar to that mentioned in the previous paragraph) independent of NK or T cells. Important through adhesion molecules such as ICAM and VCAM are upregulated in tumor vasculature, which facilitate accumulation of leukocytes and ILC3 infiltration on tumour tissue (Melo-Gonzalez and Hepworth, 2017).

#### 2.1.3 In Haematological Malignancies

During fetal development, the bone marrow contains ideal stem cells for ILC development. In adults, the liver is thought to be the main location of ILC generations (Cherrier et al., 2018). In hepatocellular cancer (HCC), the absolute global number of immune cells decreased and functional ILC1 were suppressed, which led to advanced tumor progression (de Weerdt et al., 2016; Walker et al., 2019). Recent research shows CD3ε−NK1.1 + CD62L−ILC1s, which express NKp46, induce heavy TRAIL productions, exerting anti-tumour potentials (Turchinovich et al., 2018). In chronic lymphocytic leukemia (CLL) patients, ILC count in peripheral blood greatly increased compared to control samples. Interestingly, the proportion of each type of ILCs stayed relatively unchanged in leukemia patients, suggesting all ILC cells may be taking part of cancer immunity (de Weerdt et al., 2016). Another research supports this notion as Salome’s groups were able to isolate CD16− CD127+ c-Kit− CRTH2− CD56^+^ ILC1-like populations that is, capable of producing cytokines such as TRAIL and express cytotoxic markers such as NKp30 and NKp80 for tumour lysis (Turchinovich et al., 2018). When compared to leukemia patients, tumour lysis ILC1-like cells from healthy samples upregulate all their pro-toxin markers, suggesting tumour suppression effects of leukemia in peripheral blood (Salomé et al., 2019).

### 2.2 Pro-Tumorigenic ILC Functions

#### 2.2.1 In Lung Cancers

Eomes downregulation is observed alongside development of NSCLC in NKp46 expressing ILC1s (Verma et al., 2020). Even though Eomeslo ILC1 group is phenotypically similar to that of conventional ILC1s, Eomeslo ILC1s produces lower cytotoxicity and IFN-γ levels compared with Eomeshi cells, suggesting that the low Eomes levels in ILC1s may be associated with decreased cancer immunosurveillance and early tumour suppression functions (Verma et al., 2020). Not only are ILC1 groups inefficient at protecting patients in early cancers, they are also converted into ILC3s in squamous cell carcinomas. increased ILC3 frequencies in the TME during cancer development is expected to shorten patients survival (Koh et al., 2019). In transplanted Lewis lung cancers mice model, increase in ILC2s frequency was observed and associated with type II response cytokines, such as IL-5 and IL-13. Tumor-bearing mice are fed with a vitamin A-deficient diet that led to a lower survival rate, larger tumor size, and presence of alternatively activated macrophages (AAMs) in the lung region (Cui et al., 2019).

#### 2.2.2 In Melanoma and Breast Cancer

In many cancers, IL-33 is a cancer promoting cytokine and boost Tregs to accumulate in peri-tumour areas. In breast cancer associated adipose tissue, IL-33 again stimulates Tregs to accumulate as well as malign ILC2 cells. Although both cases can be solved via stimulating ILC2 with IFN-γ, malignant ILC2 loses partially its ability to recruit nearby immune cells, as such seen in the lack of eosinophil recruitment in lung inflammation with IFN-γ stimulated ILC2 (Starkey et al., 2019). ILC2s inside malignant tumours also produce IL-13 to encourage tumour growth and metastasis in mouse models. In the presence of IL-33 produced by CD45^+^ leukocytes, ILC2 functions are further suppressed. Maintaining immunosuppression functions in the tumour milieu, presence of IL-33 increased TGF-β1 producing MDSCs and reduced IFN-γ expressing NKp46 + tumour lysing cells (Martini et al., 2010; Jovanovic et al., 2014). Increased ILC3 frequency is also associated with developing breast cancers in mice models (Irshad et al., 2017). Positive correlations between ILC frequency and likelihood of lymph node metastasis is also established (Irshad et al., 2017). Jovanovic et al.’s breast cancer mouse model, CCL21 is the main driving force of ILC3 recruitment around the tumour, inducing CXCL13 that promotes ILC3 intrastromal interaction and tumour metastatic fraction RANKL (Jovanovic et al., 2014). Furthermore, as Gao’s team reported, conventional NK cells may be transformed to CD49a + CD49b + Eomes intILC1s and CD49a + CD49b− Eomes- ILC1s during melanoma (Jovanovic et al., 2014). Their results also show that trandifferentiated intILC1s and ILC1s cannot sustain immunosurveillance functions.

#### 2.2.3 In Blood Cancers

In blood cancers, CLL and AML, the production of IL-13 from cancer cells suppresses immune functions (Gao et al., 2017). With either TNF-α or IFN-γ production deceased or interrupted, anti-tumour effects are dramatically decreased. Similarly, NOTCH and AHR signaling may be downregulated in blood cancers (Gong et al., 2013; Kannan et al., 2013). From previous research, the essential function of NOTCH and AHR in ILC development is already established (Artis and Spits, 2015). With greatly reduced ILC development and expansion in blood cancers, conversion from NK or functional ILC to non-cytotoxic ILC is observed in murine models, allowing tumours to achieve immune evasion (Gao et al., 2017). Inhibition of NOTCH or AHR also assists in dysregulation ILCs, greatly reducing effective early tumour lysing cells, such as NK cells, into non-cytotoxic ILCs in AML (Lordo et al., 2021). Though further studies are needed to examine the exact mechanisms underlying pro-tumorigenic transformation of ILCs, present studies can briefly outline the intricate interactions between ILCs and blood cancers.

## 3 ILC Shaping Tumour Microenvironment

### 3.1 ILC1 Functions

Early-stage immunosurveillance and early tumour suppression are the works of ILC1 and NK cells. NK cells, which do not express MHC-I markers, quickly respond and eliminate tumour cells through activating and inhibitory receptors on the NK cell surface (Cella et al., 2008). The resulting NK cells will secrete cytotoxins responses including IFN-γ. On the other hand, ILC1s are activated through an array of cytokines such as IL-15 from tumour cells in CRC, IL-12 or IL-18 from other immune cells. ILC1s produce IFN-γ to contribute to early tumour suppression and lysis (Fuchs et al., 2013). In many cancers, IFN-γ is a critical inhibitor for cancer cells by inducing apoptosis and upregulation of MHC-I expression, thereby activating tumour-specific immunity (Kashii et al., 1999). Alongside the secretion of interferon cytokines, detectable traces of the TNF-α are also found from IL-15 activated ILC1 cells, supporting the hypothesis that ILC1 cells suppress early-stage tumours via apoptotic lysis of tumour cells. ILC1s also interact with dendritic cells and T cells to aid invigoration of the adaptive immune system. Studies have shown stimulation of CD86 on DCs and T-lymphocytes by ILC1 cells help further build tumour-specific immunity in colorectal cancers (Dadi et al., 2016). Immune cells stimulated by ILC1s display higher tumour infiltration rates, intratumor activities, and reduction in tumor growth (Dadi et al., 2016). In chronic lymphocytic leukemia, the number of identifiable ILC1 decreases, showing immunosuppression effects of chronic blood cancer (de Weerdt et al., 2016). In acute myeloid leukemia, a remarkable increase of ILC1s and ILC1 like cells is seen compared to healthy patients (Lordo et al., 2021). Put together, ILC1s produce mainly IFN-γ to aid early tumour suppression and help activate tumour specific immunity (Figure 1).

### 3.2 ILC2 Function

During the mid and later stages of cancer development, ILC2s main functions are to infiltrate the tumour and to produce cytokines to attract other immune identities. GATA3 is crucial for all ILC2 cells. GATA3 gene knockout significantly reduced the cytokine profile of ILC2 cells and its ability to attract immune cells (Motomura et al., 2014; Long et al., 2018). Interleukin-33 is especially crucial for ILC2 function. Alongside being secreted by epithelium and endothelium, damaged cells also secrete IL-33 to activate ILC2s, attracting CD8^+^ T cells and shaping the tumour microenvironment (Li et al., 2018). Recent studies involving T cell and ILC interaction in immunodeficient mice also revealed that spleen resident ILC2s help antigen-presenting cells (APCs) cross prime CD8^+^ tumour infiltrating T cells and central memory B cells (Moral et al., 2020).

In murine PDAC, recombinant IL-33 also enhances ILCs proliferation and CCL5 production, which recruits more CD103 + DCs that primes of CD8^+^ T cells. Local activation of CD8^+^ T cells also directly mediates Th1 and Th2 activity to recruit other tumour-specific immune cells (Bresler et al., 2021). In humanized murine models, ILC2 activates CD4^+^ DC and helps prime CD8^+^ T cells mainly in local drain lymph nodes and less in secondary lymph structures such as the spleen (Salimi et al., 2018). Upon immune perturbation, IL-33 activation of ILC2s is a consequence of Tregs accumulation (Molofsky et al., 2015; Monticelli et al., 2016). In short, IL-33 activated ILC2s recruits and amplifies cell response to target tumour cells. However, in metastatic tumours, where IL-33 content is decreased, the IL-33/ILC2 activation pathways cannot be triggered, and immune evasion is facilitated. Though ILC2 cells mediate this effect via the production of IL-5 as an alternate T-cell priming pathway, the efficiency and effectiveness of these pathways are currently under study. Overall, ILC2 aid tumour immunity via attraction of other immune cells and facilitating tumour infiltration (Figure 2).

### 3.3 ILC3 Function

ILC3s lead many roles in the human body’s innate cancer immunity. NCR expressing cells actively produce pro-inflammatory cytokines such as IL-22 and TNF-α and immune recruitment cytokines such as IL-8 and IL-12 (Melo-Gonzalez and Hepworth, 2017). In late-stage NSCLC, the reduced frequency of NCR + ILC3s around the lung mucosal barriers also correlated with poorer tumour infiltration from all types of immune cells (Melo-Gonzalez and Hepworth, 2017). In murine melanoma models, the release of IL-12 from various cells (including ILC1s) helps expand RORγt + ILC3 populations in local areas. Though previously thought that ILC3 function might depend on ILC1/NK class cells, studies suggest otherwise (Klose et al., 2013). Independent ILC3 functions are more correlated to the adhesion molecules on the tumour vasculature, VCAM1 (vascular adhesion molecule 1), promoting tumour infiltration from leukocytes. Similarly, other adhesion molecules such as ICAM1 also help the second cancer-related function of ILC3s: formation and maintenance of lymphoid structures in peri-tumour areas (Nussbaum et al., 2017; Shao et al., 2021). Spleen resident ILC3 (both CCR6+ and CCR6-population) displays significant anti-tumour effects when stimulated by IL-12. The IL-12/ILC3 axis is also found inside mouse models with melanoma and PDAC. Results from hyper-stimulated spleen ILC3 allow increased invasion from CD8^+^ NK, T and NKT cells (Carrega et al., 2015). CCR6+ populations are also found accumulating in new lymph structures around the tumour. To conclude, ILC3 populations can directly suppress cancer progression and promote tumour invasion from leukocytes. Lastly, ILC3s and their related cytokines. Since ILC3s heavily depend on myeloid or epithelium cells to help translate signals to them, ILC3s are sometimes stimulated by pro-inflammatory signals such as IL-23 (Hoorweg et al., 2012). IL-23 stimulation causes IL-17 to release from ILC3s, causing initial early-stage metastatic suppression but late-stage metastatic promotion. To conclude, ILC3s aid nearby immune functions via cytokines to improve host immune responses for tumour lysis abilities (Figure 2).

## 4 New Immunotherapy Treatments Involving ILC

With the emerging understanding of the various roles of ILCs in cancers and other chronic diseases, novel ILC immunotherapies are starting to form. Although these therapies are very new, they are already showing the great potential of ILCs and how they can help improve the host’s cancer immunity.

### 4.1 Immune Checkpoint Blockade Therapy

**FIGURE 3:**
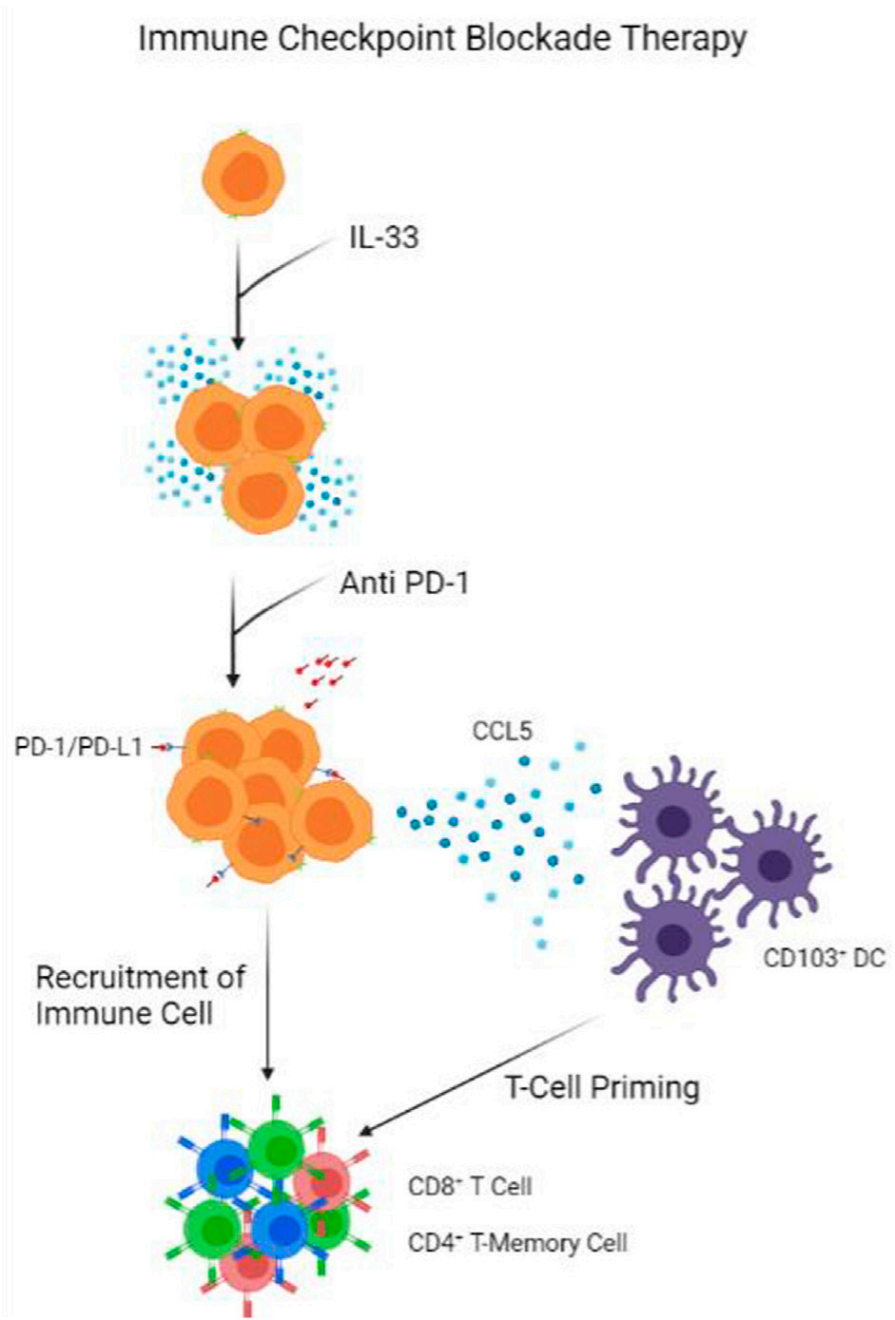
Model of Immune Checkpoint Blockade Therapy. IL-33 presence help proliferate ILC population. Anti PD-1 antibodies block PD-1 receptors on ILCs, triggering the release of CCL5 to recruit CD103^+^ DCs. 103^+^ DCs prime CD8^+^ T-cells and CD4^+^ T-Memory Cells, which are recruited by ILCs into tumour.

Immune and metabolic checkpoints, such as arginase I for ILC2s, are essential components that contribute to the functions of ILCs and the immune system (Melo-Gonzalez and Hepworth, 2017). The two most known checkpoints are the PD-1 and CTLA-4 checkpoints. A recent study involving blocking the PD-1 checkpoint mechanism unveiled novel results that boost cancer immunity in cancers (Moral et al., 2020). In murine models deficient in IL-33 production, pancreatic tumour growth and development are significantly greater than in wild-type mice. At the same time, global ILC-like cell frequency in deficient mice also reduces around 20–40 percent. However, interestingly, the difference in ILC cell count can only be observed around the tumour, not in secondary lymphoid structures or local drain lymph nodes (Moral et al., 2020). This highly localized expansion of the ILC2 population resulted in higher survival chances. On top of that, comparing the wildtype mice to IL-33 overexpression mice PDAC mice models, IL-33 overexpressing mice should have even more prolonged survival and smaller tumour volume and expansion. Furthermore, in immunodeficient mice (Rag-2 deficient), IL-33 and its recombinant variant also showed significant progress to suppress tumour growth. Concerning the PD-1 checkpoint, when Pdcd1 was knocked out, ILC2 activities increased compared to wildtype mice (Moral et al., 2020). Thus, anti-PD-1 blocking and recombinant IL-33 treatments were given to mice injected with PDAC, and on every occasion, the dual treatment effect outweighs the control or single treatment given (Moral et al., 2020) (Figure 3). Given these treatments’ abilities to enhance the patients immune responses, it should not be hard to look into some other key ILC cytokines to achieve a similar effect. A suggestion is to look at the effect of IL-5, IL-12, or IL-25 on the same murine model and how it affects survival and tumour growth as those cytokines are key for ILC functions in various cancers (Figure 2).

### 4.2 Immunoregulatory Therapies Involving ILC

**FIGURE 4:**
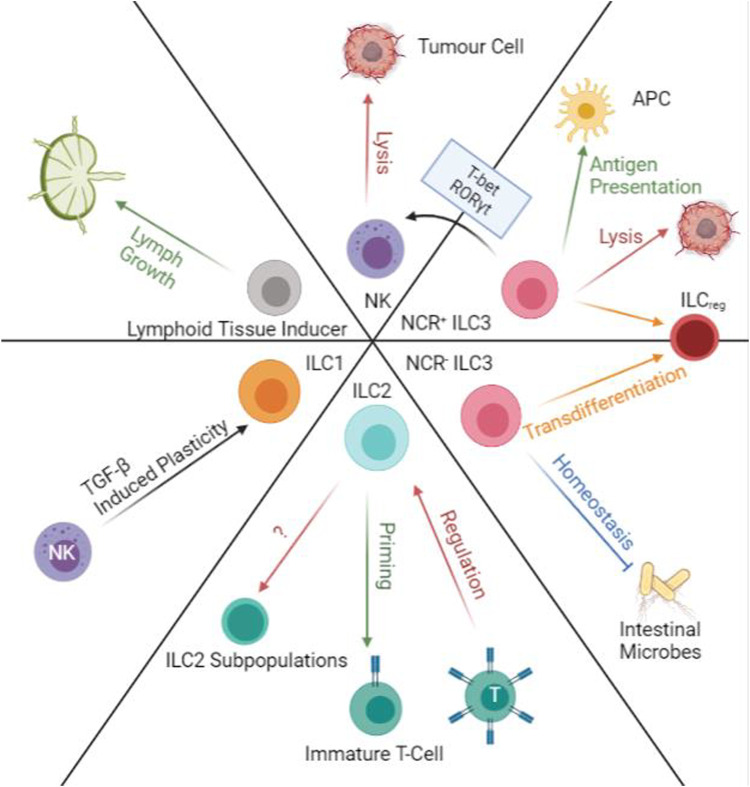
Transdifferentiation and Immunoregulatory Functions of ILCs. ILC1 and NKs have TGF-β induced plasticity that will allow ILC1s to produce IFN-γ and TNF-α. ILC2s can prime immature T-cells and at the same time are regulated by primed T-cells in lymph nodes. ILC2 populations are also able to transdifferentiate into subpopulations along with progression of cancers. NCR- ILC3s regulated intestinal microbes while NCR + ILC3s can help antigen presentation. Both populations of ILC3s are susceptible to transdifferentiation into ILCreg. NCR + ILC3s are also able to transform into NK-like cells and perform NK functions in the presence of T-bet and RORγt. NK cells can directly induce apoptosis on tumour cells. Lymphoid tissue inducers aid lymph growth.

ILCs works closely with both the adaptive and innate immune systems, recruiting APCs, DCs, and T cells to sites of inflammation or infection. Though it was previously thought that the regulatory roles of ILCs are more substantial in homeostasis, now we understand a co-regulatory relationship between ILCs and T cells (Bresler et al., 2021). In secondary lymph organs and lymph nodes, where immune cells aggregate, T cells are found to regulate the expansion of IL-33/IL-15 activated ILC2 cells. At the same time, ILC2 cells regulate the priming and maturation of T cells and other helper cells (Martin et al., 2017; Bresler et al., 2021). In immune-deficient models such as *Rag2* deficient mice, ILC populations increase in both peripheral and mesenteric lymph nodes, but global T cell priming efficiency and MCH class 1 expression are greatly reduced (Withers, 2016). A similar trend is observed regarding the expansion of ILC3 populations when T cells are lacking. Interestingly, even though the expansion of ILC2 and ILC3s increase in immunodeficient models, ILCs’ cytokine profiles decrease, suggesting functional decrease. Unfortunately, no other quantitative data detailing the exact changes of ILCs to undergo in immunodeficient models. To summarize, the co-regulatory system of T and ILC cells in lymph organs and nodes keeps both groups of cells functional. These findings alone may not be enough to form a novel treatment, but with new insights into the anti-CTLA-4 antibodies, it is possible to further look inside the relationships between ILC populations and CTLA-4 expressing cells (Pistillo, 2002). Similar to enhancing PD-1 interactions in cancers, as a suggestion, CTLA-4 interactions may also yield promising such as to help active antigen-specific immunity faster. Though currently without any published data, it is an important direction further research can chase after. Further investigation in the relationships between ILCs, macrophages, T-cells, B-cells is in strong demand to produce innovative immunotherapies (Figure 4).

### 4.3 Transdifferentiation of ILCs

ILCs are very plastic, and their functions may change according to the stimulus or local microenvironment. Previously, it was hard to map all the cell trajectories and sub-lineages of ILCs. However, thanks to current advances in cellular analysis techniques, we are slowly grasping the intricate transdifferentiation mechanisms of ILC populations. Using genomics data, the study reveals several ILC2 subpopulations and ILCreg populations in CRC (Wang et al., 2020). The three subpopulations of ILC2 seem to correlate with cancer procession from early, mid, to late stages. As the ILC2 population gets “overwhelmed” by the progression of the tumour, their secretion of anti-tumour cytokines and immune cell recruitment abilities also decline. However, the most exciting finding is the transdifferentiation from ILC3 to ILCreg populations. According to their cell trajectory data, the appearance of ILCreg populations begin in the later stages of CRC, and the population of ILC3s and ILCreg are always inversely proportional (Wang et al., 2020). Further examination also revealed that ILCregs are mostly ex-ILC3s, expressing similar surface proteins but functionally are different. Whilst ILC3s are predominantly anti-tumour, ILCreg has more pro-tumour effects, restricting tumour infiltration and tumour killing. The study suggests that ILC3 transdifferentiation is induced by the increase of TGF-β secreted by the tumour (Nussbaum et al., 2017; Wang et al., 2020). Although these ILC subpopulations are not confirmed, it is a good starting point to consider prolonging the “effective” period of ILCs before they go into transdifferentiation (Yagi et al., 2014). The intervention by cytokines and small molecules may prolong the “effective” period to create important time gaps in invasive cancer progression for the immune system to be active (Figure 4).

## 5 Future Directions

We are still discovering many unknown niche functions ILC carries out. Novel understandings of ILCs and immunotherapies involving this type of cell shows promising results for cancer patients. Unlocking ILCs’ anti-tumoral potential and suppressing their pro-tumour responses, a new field of research is gradually unveiled. Rather than directing therapies to kill tumour cells, new immunotherapies aim to boost the innate and adaptive immune responses to improve disease outcome. With PD-1 and CTLA-4 antibodies already in the commercial market, blocking immune-pathways suppressing ILC functions can help crack open alternative immune pathways that will improve survival rates and successful cure rates compared to the current standard treatments. With such diverse functions and mechanisms, understanding ILC’s many connections with other immune cells is important to develop a holistic view of the human immune system and to improve immunotherapies to prevent diseases. Unlocking further cellular mechanisms can reveal more game-changing treatments that will revolutionise cancer immunotherapies.
